# Patterns of internet and social media use in colorectal surgery

**DOI:** 10.1186/s12893-019-0518-4

**Published:** 2019-05-24

**Authors:** Leonora E. Long, Christopher Leung, Jonathan S. Hong, Caroline Wright, Christopher J. Young

**Affiliations:** 1 Sydney Medical School, University of Sydney, Camperdown, New South Wales Australia; 2 Department of Colorectal Surgery, Royal Prince Alfred Hospital, Missenden Road, Camperdown, NSW 2050 Australia; 3 Institute of Academic Surgery, Royal Prince Alfred Hospital, Camperdown, New South Wales Australia

**Keywords:** Australia, Colorectal surgery, Consumer health information, Internet, New Zealand, Social media, Surgeons

## Abstract

**Background:**

Surgeons use the Internet and social media to provide health information, promote their clinical practice, network with clinicians and researchers, and engage with journal clubs and online campaigns. While surgical patients are increasingly Internet-literate, the prevalence and purpose of searching for online health information vary among patient populations. We aimed to characterise patient and colorectal surgeon (CRS) use of the Internet and social media to seek health information.

**Methods:**

Members of the Colorectal Society of Australia and New Zealand and patients under the care of CRS at the Royal Prince Alfred Hospital, Sydney, were surveyed. Questions pertained to the types of information sought from the Internet, the platforms used to seek it, and the perceived utility of this information.

**Results:**

Most CRS spent 2–6 h per week using the Internet for clinical purposes and an additional 2–6 h per week for research. 79% preferred literature databases as an information source. CRS most commonly directed patients to professional healthcare body websites. 59% of CRS use social media, mainly for socialising or networking. Nine percent of surgeons spent > 1 h per week on social media for clinical or research purposes. 72% of surgeons have a surgical practice website.

43% of patients searched the Internet for information on their doctor, and 75% of patients sought information on their symptoms or condition. However, 25% used health-specific websites, and 14% used professional healthcare body websites. Around 84% of patients found the information helpful, and 8% found it difficult to find information on the Internet. 12% of patients used social media to seek health information.

**Conclusions:**

Colorectal surgery patients commonly find health information on the Internet but social media is not a prominent source of health information for patients or CRS.

## Background

Surgeons use the Internet and social media to provide health information, promote their clinical practice, network with clinicians and researchers, and engage with journal clubs and online campaigns [1, 2]. Younger and research-active surgeons are the most enthusiastic adopters of Internet-based communication [3]. A cross-sectional survey of Australian doctors found that while 75% engage in personal social media use, only 30.5% used email to communicate with patients and only half could offer their patients electronic forms of information [4]. Social media use by Australian surgical bodies is variable. The Royal Australasian College of Surgeons has a Facebook page, a Twitter profile, and a LinkedIn page, and encourages social media discourse by adopting hashtags for its annual congress (e.g. #ASC19). General Surgeons Australia has Facebook and LinkedIn pages but no Twitter account, and the Colorectal Surgical Society of Australia and New Zealand (CSSANZ) has no social media presence.

While surgical patients are increasingly Internet-literate, the prevalence and purpose of searching for online health information vary among patient populations [5–8]. Colorectal surgical patients may be less likely than other surgical patient populations to research their medical problem [6]. To our knowledge, there are no reports of the patterns among colorectal surgical patients in Australia of researching the Internet or social media for information on a medical condition or a particular surgeon.

We undertook survey-based research to characterise Internet and social media use among colorectal surgeons and patients.

## Methods

Surgeon surveys were mailed to members of the Colorectal Surgical Society of Australia and New Zealand (CSSANZ), with reminder surveys mailed to non-responders after three weeks. Demographics (age, gender, practice location, and training location) and the number of hours per week spent using the Internet and social media for research and clinical purposes were ascertained.

Patients under the care of colorectal surgeons at Royal Prince Alfred Hospital, Sydney, Australia were recruited by means of invitations to participate from junior doctors in the hospital treating teams and from reception staff in consultation rooms over a designated one-month period. Patients provided informed consent and completed a voluntary questionnaire of 17 items. Demographics (age, gender, level of education, and income), clinical information (elective or emergency hospital admission or reason for clinic presentation), and the number of hours spent using the Internet per week were ascertained. The types of information sought and the websites and social media platforms used were also determined.

## Results

### Surgeons

Ninety-four colorectal surgeons completed the survey, a response rate of 42%. The majority of these were male (87%), aged between 40 and 59 years (73%), practised in cities (94%) and had trained in Australia or New Zealand (68%).

**Fig. 1:**
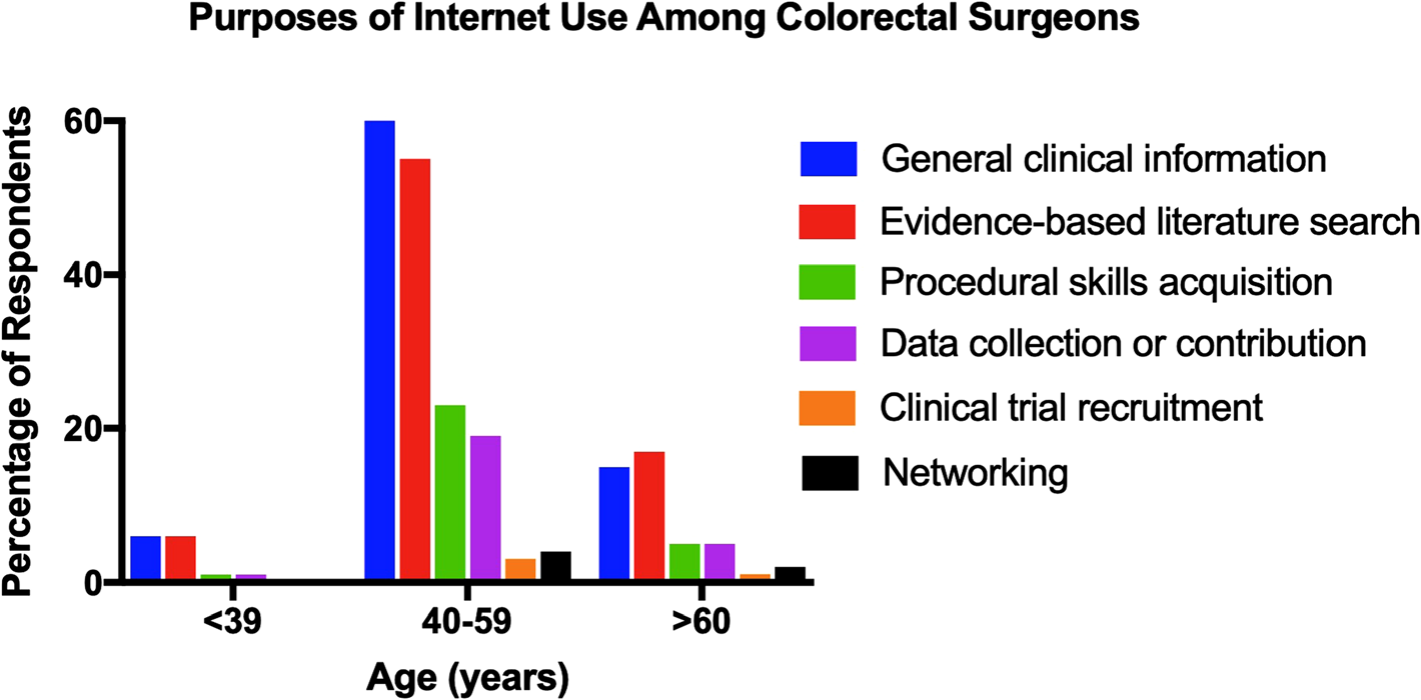
Purposes of Internet Use Among Colorectal Surgeons. *n* = 94

Around 65% of colorectal surgeons used the Internet for clinical purposes for 2–6 h per week. Internet use for research purposes was most commonly for either less than one hour (41%), or 2–6 h (50%) per week. Obtaining general clinical information (34% of respondents) or evidence-based literature search (34%) were the main purposes of surgery-related Internet use. Procedural skills acquisition (29% of respondents) and data collection or contribution (24%) were also common. The percentages of surgeons using the Internet for various purposes is illustrated according to age group in Fig. 1.

Preferred sources for online surgery-related information were evidence-based literature databases (79%) or specific journal websites (e.g. *Diseases of the Colon and Rectum*; 38%). 64% of colorectal surgeons directed patients to specific websites for further information. The most common of these were professional body websites (e.g. https://www.cssanz.org), but other examples were specific to a disease (e.g. *Crohn’s and Colitis Australia*) or condition (e.g. *Australian Council of Stoma Associations*). 72% of the surgeons surveyed had a website related to their surgical practice.

Among the 53 colorectal surgeon respondents who reported using social media for any purpose, Facebook was the most preferred platform (81%), followed by LinkedIn (47%) and Twitter (30%). However, use of social media for clinical or research purposes was limited in over 90% of surgeons to less than one hour per week or none at all. Colorectal surgeons were more likely to obtain information from than contribute to social media, with 15 respondents sharing clinical information or research findings or engaging in policy discussion. One respondent reported using social media to exchange information with postgraduate research students, and two others reported participating in social media-based journal club discussions.

### Patients

Sixty-three patients completed the survey. Patient demographics are described in Table 1.

**Table 1:** Patient demographics. Total *n* < 63 (patients declined to answer some questions)

Demographic		n
Age (years)	18–29	6
	30–39	13
	40–49	12
	50–59	13
	> 60	19
Gender	Female	40
	Male	23
Highest Education Level Completed	Primary	2
	Secondary	15
	Tertiary	29
	Postgraduate	17
Income ($ per annum)	< 40,000	18
	40,000 - 80,000	10
	80,000 - 150,000	15
	> 150,000	13

Almost one third (30%) were over 60 years of age, and 73% were educated to tertiary or postgraduate level. 58% of patients used the Internet for any purpose for 7–21 h per week, with 6% reporting less than one hour’s weekly use. Among the 39 patients (62%) who searched for information on their symptoms, 24 (75%) reported using general websites such as *Wikipedia*, 16 (25%) used health-specific sites such as *Better Health Channel,* and 9 patients (14%) reported using professional body websites. 27 patients (43%) reported searching for information on their surgeon.

Grouping patients by gender showed that men (83%) were more likely than women (72%) to search for health information. All colorectal surgical patients aged 30–39 years old searched for online health information, with this activity decreasing in successively older age brackets (58% in those over 60). All patients educated to postgraduate level searched the Internet for health-related information; this is in contrast to those educated to tertiary (79%) or primary/secondary level (53%). Similar percentages of patients presenting with abdominal pain and/or emergency hospital admissions searched the Internet for information on their symptoms to those patients presenting for a routine follow-up.

Eleven percent of patients had difficulty finding or understanding the information they sought. 84% of patients found it helpful to read health information related to their medical condition or doctor. Perceived benefits of reading information on the Internet were feeling less isolated or alone (24%), and feeling reassured about symptoms (32%) or a surgeon (14%). A reported disadvantage was feeling more concerned about symptoms (24%).

## Discussion

The low level of social media use is comparable to previous studies of colorectal surgeons, who have generally been slower to utilise Internet-based communications [1, 9]. Common reasons for low engagement in online discourse by doctors are fear of litigation or privacy concerns [4]. However, many surgeons said they used the Internet and social media for networking purposes. Moreover, the majority of colorectal surgeons surveyed had a website related to their surgical practice, so there is scope for contribution to the breadth of accurate, authoritative Internet information via this or other media, especially given the behaviour of the patients we surveyed in seeking health information.

Despite its increasing acceptance among surgeons [2], Twitter was not the most commonly used social media platform among the colorectal surgeons we surveyed. However, half of the surgeons in our sample who did use Twitter were also among the small percentage that used social media more frequently than one hour per week, suggesting a subset of avid users. These patterns reflect wider practices among doctors in Australia [4]. The #colorectalsurgery hashtag and #StrongArmSelfie colorectal cancer awareness campaign have been widely adopted outside of Australia [10, 11]. At the time of writing, the Keyhole tracker (https://keyhole.co) indicated that there were 280 #colorectalsurgery Tweets and 42 #strongarmselfie colorectal cancer awareness Tweets [11] in the last three weeks, but none from Australian Twitter users. This may also reflect patient and community factors, as doctors are often not the main contributors to social media discourse on colorectal surgical conditions [12].

Colorectal surgical patients in this study readily searched the Internet for health information. Most patients stated that they used general websites such as *Wikipedia*, and a number of patients accessed more than one type of website, including health-specific or professional body websites. However, our questionnaire did not explore the prevalence and results of search engine use among patients, which may have led to health-specific or professional body websites and thus increased the prevalence of the use of such websites.

The proportion of patients searching for online information about their surgeon was higher than in previous studies reporting that 7–26% of patients used the Internet for this purpose [3, 13, 14]. Our patients who were male, aged 30–39, or educated to postgraduate level were most likely to seek online health information. Household income [6, 14] and educational level [6] have been associated with an individual’s likelihood to research a surgical condition, and younger patients or those living further from their healthcare practitioner might be more likely to use social networking sites for any purpose [3]. We found no difference in patterns of online information seeking among subgroups of patients with differing acuity of presentation.

Further research would benefit from specific exploration of the patterns in which patients seek information, including social media sources, regarding their surgeon. We did not ask patients whether they verified their practitioner’s medical registration on the Australian Health Practitioner Regulation Authority or a professional body website. However, the majority of surveyed surgeons have clinical practice websites that include information on education credentials and membership of bodies such as CSSANZ, and these may have been viewed during patient searches for information.

The quality of colorectal surgical information websites and social media content is variable [12, 15]. While the majority of our surveyed surgeons directed patients to at least one specific website for further information, surgeons would presumably also benefit from educating patients on how to discern the quality of health-related websites utilising simple but structured criteria [16]. Furthermore, incorporating search engine optimisation into website design enhances patient exposure to information put forward by clinicians [17], and social media and web analytics may be used to direct patients to particular information sources and monitor the impact of surgeons’ online information sharing.

Limitations of the current study include the surgeon response rate (42%) and the representative nature of the sample (CSSANZ membership may not reflect all colorectal surgeons). The number of patients declining to complete the survey was not quantified, so we were unable to calculate a patient response rate. In addition, recruitment bias may have been introduced to the patient sample by selecting patients of specific colorectal surgeons with subspecialty expertise.

## Conclusions

Colorectal surgeons and patients commonly use the Internet for health information-related purposes. However, social media is not a prominent source of information for patients or colorectal surgeons. While our patients’ ease in locating and understanding Internet-based health information is in keeping with other studies [14], increased online discourse between surgeons and patients might address reported patient issues such as anxiety after reading information on the Internet.

## Abbreviations

ASC19: Annual Scientific Conference (of the Royal Australasian College of Surgeons) 2019
CSSANZ: Colorectal Surgical Society of Australia and New Zealand
